# Cognitive and mental health trajectories of COVID-19: Role of hospitalisation and long-COVID symptoms

**DOI:** 10.1192/j.eurpsy.2024.7

**Published:** 2024-02-05

**Authors:** Krupa Vakani, Martina Ratto, Anna Sandford-James, Elena Antonova, Veena Kumari

**Affiliations:** 1 Centre for Cognitive and Clinical Neuroscience, College of Health, Medicine and Life Sciences, Brunel University London, Uxbridge, UK; 2 Division of Psychology, Department of Life Sciences, College of Health, Medicine and Life Sciences, Brunel University London, Uxbridge, UK; 3 Beingwell Group, Sheffield, UK; 4 The Scale Up Collective, London, UK

**Keywords:** cognitive function, COVID-19 trajectory, intra-individual variability, long-COVID, processing speed

## Abstract

**Background:**

There is considerable evidence of cognitive impairment post COVID-19, especially in individuals with long-COVID symptoms, but limited research objectively evaluating whether such impairment attenuates or resolves over time, especially in young and middle-aged adults.

**Methods:**

Follow-up assessments (T2) of cognitive function (processing speed, attention, working memory, executive function, memory) and mental health were conducted in 138 adults (18–69 years) who had been assessed 6 months earlier (T1). Of these, 88 had a confirmed history of COVID-19 at T1 assessment (≥20 days post-diagnosis) and were also followed-up on COVID-19-related symptoms (acute and long-COVID); 50 adults had no known COVID-19 history at any point up to their T2 assessment.

**Results:**

From T1 to T2, a trend-level improvement occurred in intra-individual variability in processing speed in the COVID, relative to the non-COVID group. However, longer response/task completion times persisted in participants with COVID-19-related hospitalisation relative to those without COVID-19-related hospitalisation and non-COVID controls. There was a significant reduction in long-COVID symptom load, which correlated with improved executive function in non-hospitalised COVID-19 participants. The COVID group continued to self-report poorer mental health, irrespective of hospitalisation history, relative to non-COVID group.

**Conclusions:**

Although some cognitive improvement has occurred over a 6-month period in young and middle-aged COVID-19 survivors, cognitive impairment persists in those with a history of COVID-19-related hospitalisation and/or long-COVID symptoms. Continuous follow-up assessments are required to determine whether cognitive function improves or possibly worsens, over time in hospitalised and long-COVID participants.

## Introduction

Since the start of the coronavirus disease 2019 (COVID-19) pandemic, a vast amount of literature has acknowledged the psychological issues and cognitive disruption experienced by survivors [1–6]. Living with COVID-19 has become the new normal, yet there is still uncertainty around the longer-term effects of COVID-19 on physical and mental well-being, given marked between-study variability in the proportion of survivors reporting cognitive and mental health impairments post-acute infection [7]. In a recent review [8], 21–65% of adults with long-COVID symptoms (≥12 weeks) were found to have some level of cognitive impairment, while another review [9] reported poor mental health for up to 6 months post a COVID-19 diagnosis. It is unclear at present whether COVID-19-related cognitive impairment and psychological symptoms attenuate or resolve over time and, if so, how long after a COVID-19 diagnosis an improvement can be seen, especially in young and middle-aged adults.

Previous studies have suggested some improvement in cognitive function [10–15] and psychological well-being [16], especially at longer (≥6 months) follow-ups, but these mostly examined older adults (mean age >50 years) [10, 11, 15, 16] and focused on severely ill or hospitalised COVID-19 patients [12–15]. As these groups are likely to need longer to recover from COVID-19 and its adverse cognitive and mental health impacts, with possible co-morbidities exacerbating and/or complicating post-COVID recovery, their findings may not generalise to working-age adults in the general population. A recent study [17] involving a large sample, though again with an over-representation of middle age adults (≥50 years), showed persistent cognitive deficits at about 2 years post-infection, especially in individuals who had experienced the symptoms for ≥12 weeks and/or a severe infection, or were experiencing ongoing symptoms. Encouragingly, the sub-group of adults who self-reported a full recovery showed no such deficits [17]. There is clearly a need for further work to fully characterise the cognitive trajectory of COVID-19 in survivors with varying levels of symptoms and younger age groups.

In our recent study [18] investigating the impact of COVID-19 on cognitive function and mental health in a working-age sample (mean age: 38.70 ± 12.08), we had found a limited cognitive impact of COVID-19 diagnosis, with only intra-individual variability in processing speed being significantly increased in COVID-19 survivors, compared to non-COVID controls. There was, however, multifaceted cognitive impairment in association with long-COVID symptoms. Mental health and sleep quality were also worse in COVID-19 survivors, relative to non-COVID controls. Here, with a further assessment (6-month follow-up) of this previously assessed sample [18], we aimed to examine: (i) the longitudinal impact of COVID-19 on cognitive function, mental health, and sleep, first, on average, and then classified by COVID-19-related hospitalisation; and (ii) changes in long-COVID symptom load and their association with cognitive function, mental health and well-being at 6 months post the initial assessment. Based on previous findings [10–12, 14, 16, 19], we predicted: (i) a change towards normalisation of cognitive function, mental health, and sleep from study entry (T1) [18] to the 6-month follow-up (T2) assessments, on average, in the COVID group, relative to non-COVID group, and (ii) persistently impaired cognitive function, mental health, and sleep in participants with a history of COVID-19-related hospitalisation and/or ongoing long-COVID symptoms.

## Methods

### Participants and design

**Figure 1.**
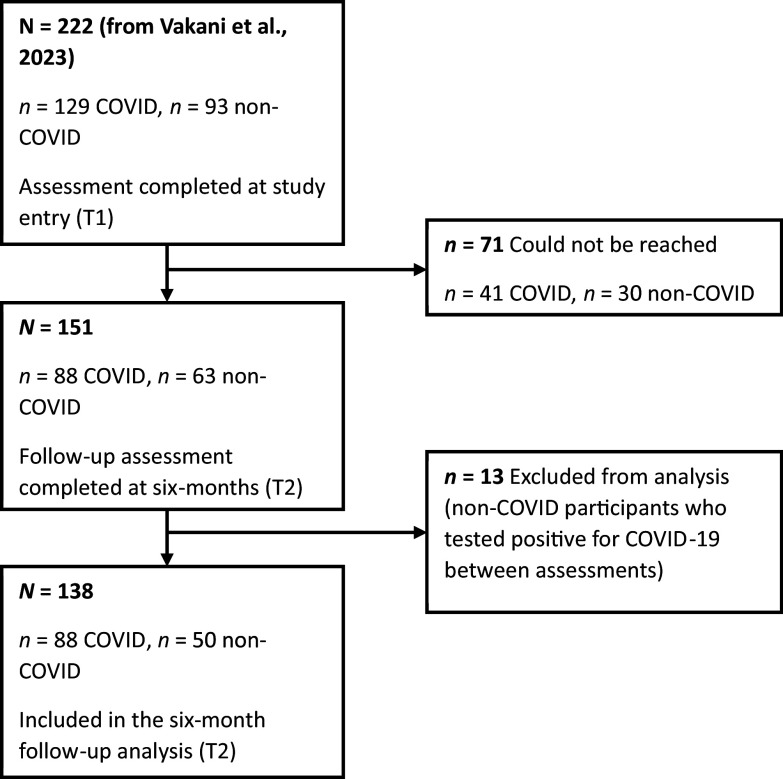
Study flowchart.

The sample consists of 138 of 222 adults who had been assessed 6 months earlier (T1; March 2021–March 2022) for our previous study investigating the cognitive impact of COVID-19 in working-age UK adults [18]. Of 222 participants (129 with and 93 without a history of COVID diagnosis) assessed at T1 [18], 71 (41 COVID, 30 non-COVID) were lost to the follow-up, and 13 non-COVID (at T1) participants were excluded due to them having tested COVID-19 positive between T1 and T2, leaving 138 participants (mean age: 39.72 ± 11.81) for this investigation (re-assessed at T2; September 2021–October 2022) (see Figure 1). Of these 138 participants (current sample), 88 had a history of COVID-19 diagnosis (14 males, 74 females; mean days since diagnosis: 459 ± 180.84; range: 163–895) (to be referred to as the “COVID group”) and 50 had no known history of COVID-19 (11 males, 39 females; to be referred to as the “non-COVID group”).

The study was approved by the College of Health, Medicine and Life Sciences Research Ethics Committee, Brunel University London (26518-A-Sep/2021-34167-1). All participants provided informed written consent and received £10 (Amazon voucher) for their time.

### Measures and procedures

As described in Vakani et al. [18], data on demographics, mental health, and sleep were collected using self-report measures administered via Qualtrics (an online survey tool), taking ˜45 minutes in total to complete. Additionally, the COVID groups were asked to detail their COVID-19 diagnosis, acute symptoms at the time of infection, subjective psychological well-being and cognitive impairment, and chronic long-COVID symptoms at both T1 and T2. Cognitive data (T1 and T2) were collected using the self-administered MyCognition [20] (MyCQ) PRO mobile application, taking ˜15 minutes to complete.

### Assessments

#### Cognitive function

The MyCQ mobile application tool (approved by the National Health Service in the UK) assesses processing speed, attention, working memory, executive function, and memory domains, using digital versions of commonly utilised neuropsychological tests validated against the Cambridge Neuropsychological Automated Test Battery [21–23]. As described previously [18], *Processing Speed* was assessed using a simple reaction time (RT) task, *Attention* using a choice RT task, *Working Memory* using the 2-back task, *Executive Function* using the Trail-Making B task, and memory was assessed using a visual recognition memory task (for further details, see Table 1).

**Table 1.** Cognitive domains, tests, and indices examined through MyCognition’s mobile application

Cognitive domains	Cognitive test	Cognitive performance indices
Processing speed	Simple reaction time (RT)	RA (% correct), average RT (ms), RT variability
Attention	Choice RT	RA (% correct), average RT (ms)
Working memory	2-Back	RA (% correct)
Executive function	Trail-making B	RA (% correct moves), total completion time (ms)
Memory	Visual recognition memory	RA (% correct)

Abbreviations: ms, milliseconds; RA, response accuracy; RT, reaction time.

#### Mental health and sleep

The following two self-report scales were used:

*The Depression, Anxiety and Stress Scale-21* [24] assessed depression, anxiety, and stress with corresponding seven-item sub-scales. Each item is rated by participants on a four-point scale according to how often in the past week it applied to them. Higher scores indicate higher levels (severity) of symptoms. Internal consistency for all sub-scales was good-to-excellent (Cronbach’s *a* ≥ 0.82) in this sample.

*Pittsburgh Sleep Quality Index* (PSQI) [25] assessed daytime dysfunction, use of sleeping medication, sleep disturbances, habitual sleep efficiency, sleep duration, sleep latency, and subjective sleep score (scores are derived for component, plus a global score). Participants respond to the PSQI items by relating them to their past month. Higher scores indicate lower sleep quality. The PSQI had an acceptable internal consistency (global score, Cronbach’s *a* = 0.76) in this sample.

### Statistical analysis

We first examined the demographic and other characteristics of study participants who provided both T1 and T2 data (*n* = 138) versus those with only T1 data (*n* = 84; not included in any further analysis), out of 222 participants from Vakani et al. [18], to determine if there were any factors associated with non-volunteering (especially in the COVID group) for T2 assessment.

Next, to examine possible changes from T1 to T2 in the COVID group (*n* = 88), relative to those in the non-COVID group (*n* = 50), we used a 2 (Group: COVID, non-COVID) × 2 (Time: T1, T2) repeated-measures analysis of variance (ANOVA), separately for each cognitive variable, with Group as a between-subjects factor and Time as a within-subjects factor. To examine possible differences in cognitive and mental health changes of hospitalised versus non-hospitalised COVID participants, we conducted 3 (Group^Hospitalisation^: ^Hospitalised^COVID, ^Non-hospitalised^COVID, non-COVID) × 2 (Time: T1, T2) repeated-measures ANOVAs; and confirmed any significant main or interaction effects after co-varying for age, given a trend-level age difference between hospitalised and non-hospitalised participants (see Results). To examine a change from T1 to T2 in total long-COVID symptom load (a sum of all symptom ratings), we ran a 2 (Hospitalisation: ^Hospitalised^COVID, ^Non-hospitalised^COVID) × 2 (Time: T1, T2) ANOVA with Hospitalisation as a between-subjects factor and Time as a within-subjects factor, co-varying for age. All ANOVAs were initially conducted with Sex entered as another between-subjects factor but Sex was then removed, as there were no main or interactive effects involving Sex, and the current sample has a relatively smaller number of males. Significant main effects and interactions from ANOVAs were followed up with the analysis of simple main effects and post hoc comparisons, as appropriate. Effect sizes, where reported, are partial eta squared (*η_p_*^2^; the proportion of variance associated with a factor). Finally, the relationship between changes (T1 to T2) in total long-COVID symptom load and cognitive function was examined using Pearson correlations.

All analyses were performed using the Statistical Package for Social Sciences (version 28; IBM, New York, USA). The data distribution on all variables met the assumptions of parametric statistical procedures. Alpha level for testing the significance of effects was maintained at *p* ≤ 0.05.

## Results

### Sample characteristics

About two-thirds (62%) of the sample with T1 assessments (*n* = 222) [18] provided T2 data (*n* = 138) (Figure 1). Fifteen (75%) of 20 participants with a history of hospitalisation at T1 also provided T2 data. There was no age difference [*t*(206) = 0.36, *p* = 0.72] between the groups with both T1 and T2 assessments and only T1 assessment. Other characteristics were also comparable for these (T1 and T2, T1 only) groups (Supplementary Table S1). COVID participants who completed both assessments versus those with only T1 assessment also had comparable demographics, COVID-related symptoms (Supplementary Tables S1 and S2), as well as cognitive and mental health characteristics (Supplementary Table S3).

For the current sample, there was no significant difference in age [*t*(136) = 1.66, *p* = 0.10] or BMI [*t*(136) = 1.66, *p* = 0.10] between the COVID (*n* = 88) and non-COVID groups (*n* = 50) (Table 2; for demographics, see Supplementary Table S4). ^Hospitalised^COVID participants (*n* = 15) had a higher prevalence of most long-COVID symptoms (Supplementary Table S2) and were also non-significantly older compared to ^Non-hospitalised^COVID participants (*n* = 73) [*t*(86) = 1.75, *p* = 0.08] (Supplementary Table S5).

**Table 2.** Comparison of T1 and T2 characteristics for the current sample (*N* = 138), classified by group

		COVID group (*n* = 88; 14 M, 74 F)		Non-COVID group (*n* = 50; 11 M, 39 F)	
		T1	T2	T1	T2
		Mean (*SD*)	Mean (*SD*)	Mean (*SD*)	Mean (*SD*)
Age (years)		40.47 (10.55)	40.97 (10.42)	37.04 (13.71)	37.52 (13.76)
BMI		28.94 (9.98)	30.13 (12.26)	26.58 (7.03)	26.99 (7.00)
		*n* (% of Total)	*n* (% of Total)	*n* (% of Total)	*n* (% of Total)
Physical health conditions	Cancer	3 (3.4%)	3 (3.4%)	0 (0%)	0 (0%)
	Diabetes	7 (8.0%)	6 (6.8%)	5 (10.0%)	3 (6.0%)
	Heart condition	4 (4.5%)	8 (9.1%)	2 (4.0%)	2 (4.0%)
	Immunosuppressed	7 (8.0%)	8 (9.1%)	0 (0%)	0 (0%)
	Kidney disease	1 (1.1%)	1 (1.1%)	0 (0%)	0 (0%)
	Liver disease	0 (0%)	0 (0%)	0 (0%)	0 (0%)
	Lung condition	18 (20.5%)	20 (22.7%)	4 (8.0%)	5 (10.0%)
	Neurological condition	5 (5.7%)	10 (11.4%)	0 (0%)	0 (0%)
	Obesity	12 (13.6%)	10 (11.4%)	5 (10.0%)	3 (6.0%)
	Organ transplantation	1 (1.1%)	1 (1.1%)	0 (0%)	0 (0%)
Mental health conditions	Anorexia nervosa	1 (1.1%)	1 (1.1%)	1 (2.0%)	1 (2.0%)
	Anxiety	38 (43.2%)	38 (43.2%)	19 (38.0%)	18 (36.0%)
	ADHD	3 (3.4%)	3 (3.4%)	1 (2.0%)	2 (4.0%)
	Depression	33 (37.5%)	32 (36.4%)	14 (28.0%)	14 (28.0%)
	Eating disorder(s)	7 (8.0%)	6 (6.8%)	2 (4.0%)	1 (2.0%)
	Insomnia	21 (23.9%)	24 (27.3%)	5 (10.0%)	6 (12.0%)
	OCD	4 (4.5%)	6 (6.8%)	2 (4.0%)	2 (4.0%)
	Panic disorder	7 (8.0%)	8 (9.1%)	5 (10.0%)	5 (10.0%)
	Personality disorder	3 (3.4%)	3 (3.4%)	1 (2.0%)	1 (2.0%)
	Phobias	6 (6.8%)	9 (10.2%)	6 (12.0%)	3 (6.0%)
	PTSD	12 (13.6%)	10 (11.4%)	3 (6.0%)	3 (6.0%)
	Psychosis	1 (1.1%)	1 (1.1%)	1 (2.0%)	1 (2.0%)
	Schizophrenia	0 (0%)	0 (0%)	0 (0%)	1 (2.0%)
	Other	2 (2.3%)	3 (3.4%)	0 (0%)	1 (2.0%)

Abbreviations: ADHD, attention–deficit hyperactivity disorder; BMI, body mass index; F, females; M, males; OCD, obsessive compulsive disorder; PTSD, post-traumatic stress disorder.

### Cognitive function: Changes from T1 to T2

#### COVID versus non-COVID participants

**Figure 2.**
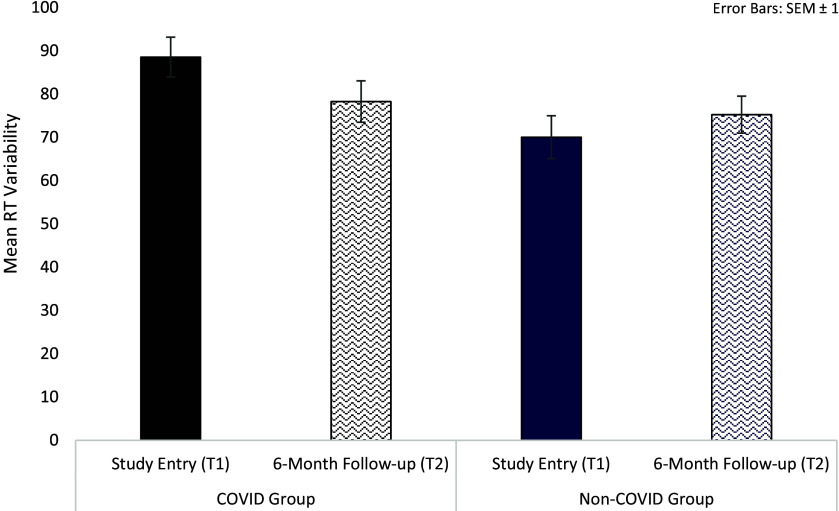
Processing speed reaction time (RT) variability in COVID and non-COVID groups at study entry (T1) and 6-month follow-up (T2).

For processing speed, we observed a significant Group × Time interaction in intra-individual RT variability [*F*(1,126) = 3.77, *p* = 0.05, *η_p_*^2^ = 0.03] (Table 3). Follow-up analysis showed significantly larger RT variability in the COVID group compared to the non-COVID group at T1 [*t*(126) = 2.63, *p* = 0.01], but not at T2 [*t*(126) = 0.44, *p* = 0.67]. From T1 to T2, there was a trend-level improvement in the COVID group [*t*(78) = 1.92, *p* = 0.06], with comparable T1 and T2 scores (i.e., no change) in the non-COVID group [*t*(48) = 0.99, *p* = 0.33] (Table 3; Figure 2).

**Table 3.** Descriptive statistics and results of the repeated-measures Group (COVID, non-COVID) × Time (T1, T2) analysis of variance (ANOVA) on cognitive measures

		COVID group (*n* = 88)		Non-COVID group (*n* = 50)		Group (COVID, non-COVID) × Time (T1, T2) ANOVA results											
		T1: Study entry	T2: 6-month follow-up	T1: Study entry	T2: 6-month follow-up	Group				Time				Group × Time			
		Mean (*SD*)	Mean (*SD*)	Mean (*SD*)	Mean (*SD*)	*F*	*df*	*p*	*η_p_*^2^	*F*	*df*	*p*	*η_p_*^2^	*F*	*df*	*p*	*η_p_*^2^
Processing speed^a^	Response accuracy (%)	95.76 (6.30)	96.71 (4.47)	95.78 (7.60)	96.41 (4.31)	0.03	1,126	0.87	*0.0*	1.65	1,126	0.20	*0.01*	0.07	1,126	0.80	*0.001*
	RT (correct responses, ms)	376.51 (80.83)	367.54 (86.94)	354.71 (79.94)	345.90 (50.58)	2.84	1,126	0.09	*0.02*	2.29	1,126	0.13	*0.02*	0.0	1,126	0.99	*0.0*
	RT variability (SD of RT)	88.54 (40.89)	78.27 (42.53)	70.04 (34.67)	75.24 (29.97)	3.51	1,126	0.06	*0.03*	0.41	1,126	0.53	*0.003*	3.77	1,126	**0.05**	*0.03*
Attention^b^	Response accuracy (%)	95.36 (8.79)	95.02 (9.42)	97.71 (4.48)	95.64 (6.38)	1.55	1,123	0.22	*0.01*	2.06	1,123	0.15	*0.02*	1.05	1,123	0.31	*0.01*
	RT (correct responses, ms)	490.53 (92.15)	494.69 (114.00)	463.52 (92.97)	450.40 (87.67)	4.67	1,123	**0.03**	*0.04*	0.35	1,123	0.56	*0.003*	1.29	1,123	0.26	*0.01*
Working memory^c^	Response accuracy (%)	92.44 (8.48)	93.53 (7.03)	92.98 (7.83)	94.31(6.14)	0.33	1,132	0.57	*0.002*	2.79	1,132	0.10	*0.02*	0.03	1,132	0.87	*0.0*
Executive function^d^	Accuracy (%)	94.48 (7.48)	94.32 (9.02)	95.11 (8.50)	92.56 (12.74)	0.19	1,134	0.66	*0.001*	1.72	1,134	0.19	*0.01*	1.34	1,134	0.25	*0.01*
	Completion time (ms)	33692.22 (22321.50)	32263.90 (23740.74)	29556.16 (9761.48)	33450.29 (31759.02)	0.17	1,134	0.68	*0.001*	0.37	1,134	0.54	*0.003*	1.74	1,134	0.19	*0.01*
Memory^e^	Recognition accuracy (%)	89.95 (9.11)	92.05 (6.38)	92.30 (7.50)	92.56 (6.17)	1.60	1,135	0.21	*0.01*	2.78	1,135	0.10	*0.02*	1.71	1,135	0.19	*0.01*

Abbreviations: ms, milliseconds; RT, reaction time.

Sample size reduced ^a^by 10 (9 COVID, 1 non-COVID), ^b^by 13 (11 COVID, 2 non-COVID), ^c^by 4 (1 COVID, 3 non-COVID), ^d^by 2 (1 COVID, 1 non-COVID), ^e^by 1 (COVID).

For attention, there was only a main effect of Group in RTs [*F*(1,123) = 4.67, *p* = 0.03, *η_p_*^2^ = 0.04], showing slower RTs on both occasions in the COVID group, relative to the non-COVID group (Table 3).

For working memory, executive function, and memory tasks, no significant main effects or interactions were found.

#### The influence of COVID-19-related hospitalisation history

For processing speed, there were main effects of Group^Hospitalisation^ for both average RTs [*F*(2,125 = 3.71, *p* = 0.03, *η_p_*^2^ = 0.06] and RT variability [*F*(2,125 = 3.33, *p* = 0.04, *η_p_*^2^ = 0.05]. Follow-up analysis of RTs showed significantly larger RTs in the ^Hospitalised^COVID group relative to the ^Non-hospitalised^COVID group [*F*(1,77) = 3.87, *p* = 0.05, *η_p_*^2^ = 0.05; age co-varied: *F*(1,76) = 3.36, *p* = 0.07, *η_p_*^2^ = 0.04], as well as the non-COVID group [*F*(1,60 = 8.44, *p* = 0.005, *η_p_*^2^ = 0.12; age co-varied: *F*(1,59 = 6.76, *p* = 0.01, *η_p_*^2^ = 0.10]. The ^Non-hospitalised^COVID and non-COVID groups did not differ from each other [*F*(1,113 = 1.24, *p* = 0.27, *η_p_*^2^ = 0.01] (Table 4). Follow-up analysis of processing speed RT variability showed that the ^Hospitalised^COVID group had larger RT variability compared to the non-COVID group [*F*(1,60 = 8.62, *p* = 0.005, *η_p_*^2^ = 0.01; age co-varied: *F*(1,59 = 6.83, *p* = 0.01, *η_p_*^2^ = 0.10] but not the ^Non-hospitalised^COVID group [*F*(1,77) = 2.63, *p* = 0.11, *η_p_*^2^ = 0.03; age co-varied: *F*(1,76) = 2.46, *p* = 0.12, *η_p_*^2^ = 0.03] (Table 4). There was no significant difference between the ^Non-hospitalised^COVID and non-COVID groups [*F*(1,113 = 1.80, *p* = 0.18, *η_p_*^2^ = 0.02].

**Table 4.** Descriptive statistics and results of the repeated-measures Group^Hospitalisation^ (^Hospitalised^COVID, ^Non-hospitalised^COVID, non-COVID) × Time (T1, T2) analysis of variance (ANOVA) on cognitive measures

			^Hospitalised^COVID group (*n* = 15)		^Non-hospitalised^COVID group (*n* = 73)		Group ^Hospitalisation^ (^Hospitalised^COVID, ^Non-hospitalised^COVID, non-COVID) × Time (T1, T2) ANOVA results											
			T1: Study entry	T2: 6-month follow-up	T1: Study entry	T2: 6-month follow-up	Group^Hospitalisation^				Time				Group^Hospitalisation^ × Time			
			Mean (*SD*)	Mean (*SD*)	Mean (*SD*)	Mean (*SD*)	*F*	*df*	*p*	*η*_*p*_^*2*^	*F*	*df*	*p*	*η*_*p*_^*2*^	*F*	*df*	*p*	*η*_*p*_^*2*^
Processing speed^a^	Response accuracy (%)	Non-COVID group presented in Table 3	94.99 (9.06)	95.45 (5.50)	95.92 (5.68)	96.96 (4.24)	0.38	2,125	0.68	*0.01*	0.88	1,125	0.35	*0.01*	0.07	2,125	0.93	*0.001*
	RT (correct responses, ms)		417.46 (94.65)	401.77 (94.16)	368.44 (76.06)	360.80 (84.58)	3.71	2,125	**0.03**	*0.06*	2.19	1,125	0.14	*0.02*	0.08	2,125	0.92	*0.001*
	RT variability (SD of RT)		99.92 (35.62)	94.77 (35.87)	86.30 (41.73)	75.02 (43.21)	3.33	2,125	**0.04**	*0.05*	0.58	1,125	0.45	*0.01*	1.98	2,125	0.14	*0.03*
Attention^b^	Response accuracy (%)		94.54 (7.03)	92.85 (11.01)	95.51 (9.12)	95.42 (9.15)	1.15	2,122	0.32	*0.02*	1.48	1,122	0.23	*0.01*	0.68	2,122	0.51	*0.01*
	RT (correct responses, ms)		554.17 (75.13)	576.67 (123.67)	478.78 (90.63)	479.55 (106.36)	7.54	2,122	**0.001**	*0.11*	0.13	1,122	0.72	*0.001*	0.99	2,122	0.38	*0.02*
Working memory^c^	Response accuracy (%)		90.94 (7.37)	94.03 (4.22)	92.75 (8.71)	93.43 (7.50)	0.22	2,131	0.81	*0.003*	3.98	1,131	**0.05**	*0.03*	0.58	2,131	0.56	*0.01*
Executive function^c^	Accuracy (%)		91.36 (11.78)	92.72 (12.54)	95.13 (6.16)	94.66 (8.17)	1.06	2,133	0.35	*0.02*	0.20	1,133	0.65	*0.002*	0.83	2,133	0.44	*0.01*
	Completion time (ms)		44595.93 (34257.70)	47056.93 (44537.02)	31420.61 (18486.50)	29182.01 (15352.91)	3.91	2,133	**0.02**	*0.06*	0.33	1,133	0.57	*0.002*	1.13	2,133	0.33	*0.02*
Memory^c^	Recognition accuracy (%)		88.06 (11.49)	90.63 (6.55)	90.34 (8.58)	92.35 (6.35)	1.42	2,134	0.25	*0.02*	3.63	1,134	0.06	*0.03*	0.88	2,134	0.42	*0.01*

Sample size reduced ^a^by 9 (2 Hospitalised, 7 non-hospitalised), ^b^by 11 (3 Hospitalised, 8 non-hospitalised), ^c^by 1 (non-hospitalised).

Abbreviations: ms, milliseconds; RT, reaction time.

For attention task RTs, there was a main effect of Group^Hospitalisation^ [*F*(2,122 = 7.54, *p* = 0.001, *η_p_*^2^ = 0.11], with larger RTs in the ^Hospitalised^COVID group relative to the ^Non-hospitalised^COVID group [*F*(1,75) = 9.60, *p* = 0.003, *η_p_*^2^ = 0.11; age co-varied: *F*(1,74) = 10.01, *p* = 0.002, *η_p_*^2^ = 0.12] as well as the non-COVID group [*F*(1,58 = 15.95, *p* < 0.001, *η_p_*^2^ = 0.22; age co-varied: *F*(1,57 = 14.23, *p* < 0.001, *η_p_*^2^ = 0.20]. There was no difference between the ^Non-hospitalised^COVID and non-COVID groups [*F*(1,111 = 1.82, *p* = 0.18, *η_p_*^2^ = 0.02] (Table 4).

For working memory (RA, %), there was only a marginally significant main effect of Time [*F*(1,131) = 3.98, *p* = 0.05, *η_p_*^2^ = 0.03; higher RA at T2 than T1], which became non-significant after co-varying for age [*F*(1,130) = 3.09, *p* = 0.08, *η_p_*^2^ = 0.02] (Table 4).

For executive function, there was a main effect of Group^Hospitalisation^ in task completion time (ms) [*F*(2,133 = 3.91, *p* = 0.02, *η_p_*^2^ = 0.06], explained by longer completion time (across T1 and T2) in ^Hospitalised^COVID group relative to both the ^Non-hospitalised^COVID [*F*(1,85) = 6.72, *p* = 0.011, *η_p_*^2^ = 0.07; age co-varied: *F*(1,84) = 6.11, *p* = 0.02, *η_p_*^2^ = 0.07] and non-COVID [*F*(1,62 = 4.15, *p* = 0.046, *η_p_*^2^ = 0.06; age co-varied: *F*(1,61 = 2.30, *p* = 0.14, *η_p_*^2^ = 0.04] groups. There was no difference between the ^Non-hospitalised^COVID and non-COVID groups [*F*(1,119 = 0.61, *p* = 0.69, *η_p_*^2^ = 0.001].

For memory tasks, no significant main effects or interactions were found (Table 4).

### Mental health and sleep: Changes from T1 to T2

#### COVID versus non-COVID participants

There were significant main effects of Group in depression [*F*(1,136) = 5.09, *p* = 0.03, *η_p_*^2^ = 0.04], anxiety [*F*(1,136) = 5.89, *p* = 0.02, *η_p_*^2^ = 0.04], and overall sleep quality [*F*(1,136) = 26.49, *p* < 0.001, *η_p_*^2^ = 0.16]. The COVID group had significantly higher depression and anxiety and lower sleep quality (PSQI) compared to the non-COVID group. Additionally, there was a main effect of Time for depression [*F*(1,136) = 4.73, *p* = 0.03, *η_p_*^2^ = 0.03] explained by lower depression at T2 relative to T1 in both groups (Table 5). No significant effects (only trends) were found for stress.

**Table 5.** Descriptive statistics and results of the repeated-measures Group (COVID, non-COVID) × Time (T1, T2) analysis of variance (ANOVA) on mental health and sleep measures

	COVID group (*n* = 88)		Non-COVID group (*n* = 50)		Group (COVID, non-COVID) × Time (T1, T2) ANOVA results											
	T1: Study entry	T2: 6-month follow-up	T1: Study entry	T2: 6-month follow-up	Group				Time				Group × Time			
	Mean (*SD*)	Mean (*SD*)	Mean (*SD*)	Mean (*SD*)	*F*	*df*	*p*	*η*_*p*_^*2*^	*F*	*df*	*p*	*η*_*p*_^*2*^	*F*	*df*	*p*	*η*_*p*_^*2*^
Mental health (DASS-21)																
Depression	14.11 (10.50)	11.61 (10.78)	9.36 (9.69)	8.76 (9.84)	5.09	136	**0.03**	*0.04*	4.73	136	**0.03**	*0.03*	1.78	136	0.19	*0.01*
Anxiety	10.59 (8.75)	10.41 (9.25)	7.04 (7.56)	7.08 (8.13)	5.89	136	**0.02**	*0.04*	0.02	136	0.90	*0.0*	0.04	136	0.84	*0.0*
Stress	14.70 (9.26)	12.95 (9.83)	13.28 (10.19)	12.76 (10.15)	0.25	136	0.62	*0.002*	3.22	136	0.08	*0.02*	0.95	136	0.33	*0.01*
Sleep quality (PSQI)																
Global score^a^	9.95 (3.70)	9.64 (4.00)	6.54 (3.25)	6.76 (3.68)	26.49	136	**<0.001**	*0.16*	0.04	136	0.84	*0.0*	1.19	136	0.28	*0.01*

DASS-21, The Depression, Anxiety and Stress Scale-21; PSQI, Pittsburgh Sleep Quality Index.

Higher scores indicate higher levels of depression, anxiety and stress. Higher scores indicate poor sleep quality.

a The Group Effect was present on all PSQI sub-components, indicating poorer sleep quality in the COVID compared to the non-COVID group.

#### The influence of COVID-19-related hospitalisation history

For depression, there was a main effect of Group^Hospitalisation^ [*F*(2,134) = 2.99, *p* = 0.05, *η_p_*^2^ = 0.04], with no difference between the ^Non-hospitalised^COVID and ^Hospitalised^COVID groups [*F*(1,86) = 0.19, *p* = 0.67, *η_p_*^2^ = 0.002] but a trend for higher depression in both ^Non-hospitalised^COVID [*F*(1,121) = 3.99, *p* = 0.05, *η_p_*^2^ = 0.03; age co-varied: *F*(1,120) = 4.35, *p* = 0.04, *η_p_*^2^ = 0.04] and ^Hospitalised^COVID [*F*(1,63) = 3.69, *p* = 0.06, *η_p_*^2^ = 0.06; age co-varied: *F*(1,62) = 3.65, *p* = 0.06, *η_p_*^2^ = 0.06] COVID groups, relative to the non-COVID group (Table 6). There was also a trend-level Group^Hospitalisation^ × Time interaction [*F*(2,134) = 2.67, *p* = 0.07, *η_p_*^2^ = 0.04], explained by a significant reduction (T1 to T2) in depression in the ^Non-hospitalised^COVID group [*t*(72) = 3.31, *p* = 0.001], but no significant change in the ^Hospitalised^COVID [*t*(14) = 0.68, *p* = 0.51] or non-COVID [*t*(49) = 0.54, *p* = 0.59] groups (Table 6).

**Table 6.** Descriptive statistics (non-COVID group presented in Table 5) and results of the repeated-measures Group^Hospitalisation^ (^Hospitalised^COVID, ^Non-hospitalised^COVID, non-COVID) × Time (T1, T2) analysis of variance (ANOVA) on mental health and sleep measures

		^Hospitalised^COVID group (*n* = 15)		^Non-hospitalised^COVID group (*n* = 73)		Group^Hospitalisation^ (Hospitalised COVID, ^Non-hospitalised^COVID, non-COVID) × Time (T1, T2) ANOVA results											
		T1: Study entry total	T2: 6-month follow-up total	T1: Study entry total	T2: 6-month follow-up total	Group^Hospitalisation^				Time				Group × Time			
		Mean (*SD*)	Mean (*SD*)	Mean (*SD*)	Mean (*SD*)	*F*	*df*	*p*	*η*_*p*_^*2*^	*F*	*df*	*p*	*η*_*p*_^*2*^	*F*	*df*	*p*	*η*_*p*_^*2*^
Mental health (DASS-21)	Non-COVID group presented in Table 5																
Depression		13.33 (7.43)	14.40 (7.38)	14.27 (11.06)	11.04 (11.30)	2.99	2,134	**0.05**	*0.04*	1.37	1,134	0.24	*0.01*	2.67	2,134	0.07	*0.04*
Anxiety		11.60 (7.49)	10.80 (8.06)	10.38 (9.02)	10.33 (9.52)	4.13	2,134	**0.02**	*0.06*	0.02	1,134	0.88	*0.0*	0.10	2,134	0.90	*0.002*
Stress		17.20 (7.44)	18.27 (8.81)	14.19 (9.55)	11.86 (9.72)	2.79	2,134	0.07	*0.04*	1.90	1,134	0.17	*0.01*	1.84	2,134	0.16	*0.03*
Sleep quality (PSQI)																	
Global score		10.80 (4.06)	10.93 (3.85)	9.78 (3.63)	9.37 (4.00)	13.28	2,134	**<0.001**	*0.17*	0.74	1,134	0.39	*0.01*	0.79	2,134	0.46	*0.01*

DASS-21, The Depression, Anxiety and Stress Scale-21; PSQI, Pittsburgh Sleep Quality Index.

Higher scores indicate higher levels of depression, anxiety and stress. Higher scores indicate poor sleep quality.

For anxiety, there was a main effect of Group^Hospitalisation^ [*F*(2,134) = 4.13, *p* = 0.02, *η_p_*^2^ = 0.06], with both ^Hospitalised^COVID [*F*(1,63) = 3.93, *p* = 0.05, *η_p_*^2^ = 0.06; age co-varied: *F*(1,62) = 3.89, *p* = 0.05, *η_p_*^2^ = 0.06] and ^Non-hospitalised^COVID [*F*(1,121) = 4.85, *p* = 0.03, *η_p_*^2^ = 0.04; age co-varied: *F*(1,120) = 6.23, *p* = 0.01, *η_p_*^2^ = 0.05] groups showing higher anxiety relative to the non-COVID group (Table 6). No difference was found between the ^Non-hospitalised^COVID and ^Hospitalised^COVID groups [*F*(1,86) = 0.12, *p* = 0.73, *η_p_*^2^ = 0.001].

Finally, there was a main effect of Group^Hospitalisation^ in sleep quality [*F*(2,134 = 13.28, *p* < 0.001, *η_p_*^2^ = 0.17], with a lower sleep quality in both ^Non-hospitalised^COVID [*F*(1,121 = 21.69, *p* < 0.001, *η_p_*^2^ = 0.15; age co-varied: *F*(1,120 = 21.05, *p* < 0.001, *η_p_*^2^ = 0.15] and ^Hospitalised^COVID [*F*(1,63 = 18.60, *p* < 0.001, *η_p_*^2^ = 0.23; age co-varied: *F*(1,62 = 15.29, *p* < 0.001, *η_p_*^2^ = 0.20] groups, relative to the non-COVID group. The ^Non-hospitalised^COVID and ^Hospitalised^COVID groups did not differ from each other [*F*(1,86 = 1.64, *p* = 0.20, *η_p_*^2^ = 0.02] (Table 6).

### *Long-COVID symptoms: Change from T1 to T*2 *in COVID participants*

**Figure 3.**
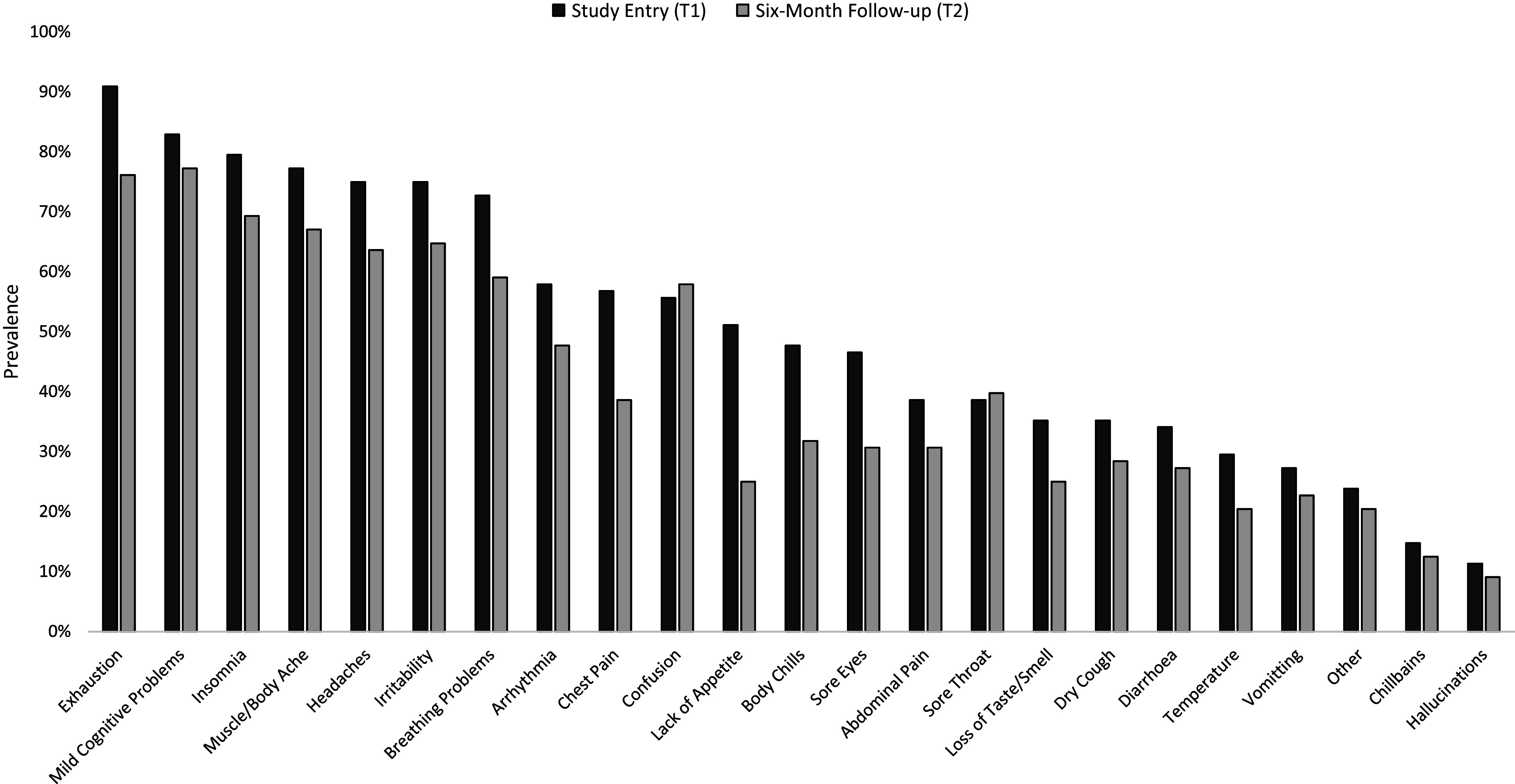
Prevalence of self-reported chronic COVID-19 (long-COVID) symptoms in the current sample (*n* = 82 of 88 provided data) at study entry (T1) and the 6-month follow-up (T2).

A similar pattern of self-reported long-COVID symptoms, with exhaustion and mild cognitive problems being the most prevalent, was seen at T1 and T2 (Figure 3), especially in the ^Hospitalised^COVID group (Supplementary Table S2).

**Figure 4.**
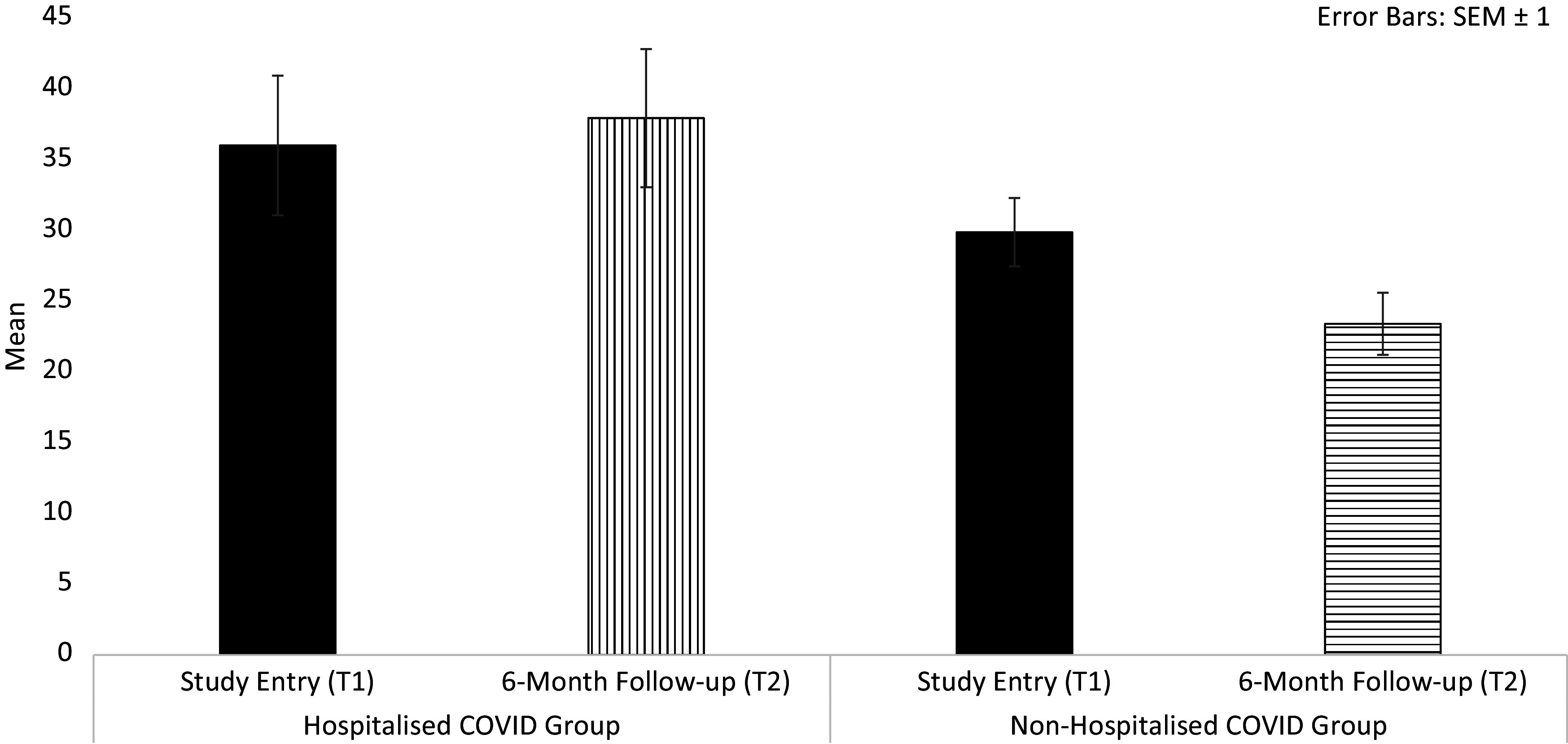
Total long-COVID symptom load in COVID participants, classified by hospitalisation history.

Total long-COVID symptom load showed a main effect of Time [*F*(1,79) = 4.86, *p* = 0.03, *η_p_*^2^ = 0.06) and, importantly, a Hospitalisation × Time interaction [*F*(1,79) = 5.18, *p* = 0.03, *η_p_*^2^ = 0.06], explained by a marked reduction (T1 to T2) in symptom load in ^Non-hospitalised^COVID [*t*(67) = 5.25, *p* < 0.001] but not in ^Hospitalised^COVID participants [*t*(13) = 0.49, *p* = 0.63] (Figure 4). Long-COVID symptom load did not correlate significantly with the number of days since diagnosis [*r*(82) = 0.16, *p* = 0.15].

### Long-COVID symptoms, cognitive indices, and mental health: Inter-relationships

Higher long-COVID symptom load was associated with poorer performance on most cognitive indices (Table 7). The reduction in symptom load from T1 to T2 correlated significantly with an improvement in executive function RA (%) when examined across all COVID participants (*p* = 0.03), and in ^Non-hospitalised^COVID participants (*p* = 0.003) (Table 7).

**Table 7.** Associations (Pearson’s *r*) of total long-COVID symptom load (at T1 and T2, and the change from T1 to T2) with cognitive function and mental health (at T1 and T2, and the change from T1 to T2)

		Correlations of total long-COVID symptom load with cognitive function, mental health, and sleep						Correlations between decrease in total long-COVID symptom load from T1 to T2^a^ and improvement in cognitive function and mental health								
		At T1			At T2^a^			All COVID participants			Hospitalised group			Non-Hospitalised group		
		*r*	*p*	*n*	*r*	*p*	*n*	*r*	*p*	*n*	*r*	*p*	*n*	*r*	*p*	*n*
Processing speed	Response accuracy %	−0.21	0.06	80	−0.10	0.40	78	0.06	0.59	73	0.26	0.41	12	0.002	0.99	61
	RT correct responses, ms	0.29	**0.01**	80	0.44	**<0.001**	78	−0.11	0.34	73	0.07	0.82	12	−0.16	0.22	61
	RT variability SD of RT	0.19	0.09	80	0.42	**<0.001**	78	−0.07	0.53	73	0.14	0.67	12	−0.13	0.31	61
Attention	Response accuracy %	−0.21	0.07	80	−0.32	**0.01**	77	0.21	0.08	71	0.39	0.23	11	0.18	0.18	60
	RT correct responses, ms	0.31	**0.01**	80	0.53	**<0.001**	77	0.00	1.00	71	0.16	0.64	11	−0.06	0.64	60
Working memory	Response accuracy %	−0.17	0.11	87	−0.23	**0.04**	82	−0.11	0.32	81	0.01	0.99	14	−0.12	0.34	67
Executive function	Accuracy %	−0.27	**0.01**	88	−0.21	0.06	81	0.24	**0.03**	81	−0.001	1.00	14	0.36	**0.003**	67
	Completion time, ms	0.31	**0.003**	88	0.37	**0.001**	81	0.09	0.41	81	−0.31	0.29	14	0.20	0.11	67
Memory	Recognition accuracy %	−0.30	**0.01**	87	−0.45	**<0.001**	81	0.18	0.12	81	0.40	0.16	14	0.10	0.42	67
Mental health (DASS-21)	Depression	0.28	**0.01**	88	0.41	**<0.001**	82	0.32	**0.003**	82	0.66	**0.01**	14	0.21	0.08	68
	Anxiety	0.54	**<0.001**	88	0.56	**<0.001**	82	0.42	**<0.001**	82	0.62	**0.02**	14	0.42	**<0.001**	68
	Stress	0.33	**0.002**	88	0.36	**0.001**	82	0.30	**0.01**	82	0.50	0.07	14	0.20	0.11	68
Sleep quality (PSQI)	Global score	0.39	**<0.001**	88	0.46	**<0.001**	82	0.30	**0.01**	82	0.36	0.20	14	0.28	**0.02**	68

Abbreviations: ms, milliseconds; RT, reaction time.

a Long-COVID data not available for 6 participants (1 hospitalised, 5 non-hospitalised).

Across all participants, the reduction in long-COVID symptom load also correlated with a reduction in depression (*p* = 0.003), anxiety (*p* < 0.001), stress (*p* = 0.01), and improved sleep quality (*p* = 0.01); these associations were generally stronger in ^Hospitalised^COVID (*r* values 0.36 to 0.66) relative to ^Non-hospitalised^COVID participants (*r* values 0.20 to 0.42) (Table 7). Improved sleep quality correlated with an improvement in memory (*r* = 0.19, *p* = 0.03); other mental health/sleep and cognition changes associations, though in the expected direction, were non-significant (Supplementary Table S6).

## Discussion

This investigation focused on charting the cognitive and mental health trajectories of COVID-19 in a UK adult sample (≤69 years) that had been assessed 6 months earlier (T1) [18]. The findings showed: (i) a trend-level improvement (from T1 to T2) in processing speed RT variability but a continued slowing on the attention task (longer RTs) in the COVID, relative to the non-COVID group; (ii) within the COVID group, poorer cognitive function (processing speed, attention, executive function) in previously hospitalised, relative to non-hospitalised, participants on both occasions of testing (T1, T2); (iii) higher depression and anxiety, and reduced sleep quality in the COVID group, relative to the non-COVID group, at both T1 and T2, though an improvement in depression was visible in non-hospitalised COVID participants; (iv) reduced overall long-COVID symptom load at T2 compared to T1, particularly in non-hospitalised COVID participants (only a non-significant reduction in hospitalised COVID participants); (v) association between higher long-COVID symptom load and poorer performance on most cognitive indices; (vi) an association between reduced long-COVID symptom load and improved executive function at T2, again observed only in non-hospitalised COVID participants; and (vii) medium-sized associations between reduced long-COVID symptom load and improved mental health and well-being.

Regarding the impact of COVID-19 on cognitive function, in our previous study involving this working-age sample [18] the only cognitive variable to show a robust adverse impact of COVID-19 (regardless of hospitalisation history) was intra-individual variability in processing speed RTs, with larger RT variability in COVID-19 survivors compared to both non-COVID controls and their own pre-pandemic level (sub-sample for whom such data were available). The present investigation, encouragingly, demonstrated a trend towards normalisation (from T1 to T2) in this measure and thus suggested, on average, only a limited and possibly reversible adverse cognitive effects of COVID-19 in a working-age population. However, participants who had required COVID-19 hospitalisation showed continued cognitive impairment, a finding which is well documented in the literature, with hospitalisation status significantly impacting cognitive function and the speed of any possible recovery months after initial infection and hospitalisation [13, 26–31]. Our findings are also consistent with earlier findings of Del Brutto et al. [11] who observed an improvement towards normalisation in Montreal Cognitive Assessment scores at 18 months post-infection in older adults (mean age: 62.7 years) who had a history of mild COVID-19 and no hospitalisation and had shown a significant impairment when assessed earlier at 6 months post-infection. Their findings, taken together with ours, suggest cognitive improvement towards normalisation in COVID-19 survivors, especially without COVID-19-related hospitalisation, and that this recovery may occur relatively earlier (6–12 months post-COVID-19) in younger/working-age samples. Hospitalised COVID participants in our and other samples may show persistent cognitive impairment as a consequence of COVID-19-related structural and/or functional changes in the brain [32, 33], which needs to be explored further.

Regarding total long-COVID symptom load, a significant reduction was observed from T1 to T2, which significantly correlated with improved executive function only in non-hospitalised COVID participants, again suggesting a stronger/faster recovery in those without a hospitalisation history. However, for the majority of the sample, regardless of hospitalisation history, various self-reported long-COVID symptoms were still present at T2, with sizeable associations between long-COVID symptom load and cognitive function, in line with previous findings [34, 35].

Mental health and sleep were still impacted at T2 in COVID-19 survivors, irrespective of hospitalisation history, though depression was lower at T2 than T1 in those without COVID-19-related hospitalisation. Notably, sleep appeared to be the most impacted. Interestingly, recent findings show that people with a COVID-19 history are more likely to be a late/evening chronotype, compared to those with no known history of COVID-19 [36], and late chronotype itself has been associated with poor quality of sleep [37–39]. There are also suggestions that the lockdowns resulted in delayed chronotype due to the altered social schedule, such as, reduced exposure to sunlight coupled together with longer and later sleeping patterns, which can all contribute to reduced quality of sleep [37, 40, 41]. It is possible that those with a history of COVID-19 were more impacted by subsequent lockdowns and shifted more towards eveningness and consequently poor sleep quality.

The strengths of this follow-up study include: (i) the response rate was reasonable with about two-thirds of the original sample [18] available for this investigation, and (ii) the current sample was representative of the original sample. Nonetheless, the limitation of relying on self-report for COVID-19-related information inherent to our earlier study [18] also applies to this study. Despite this limitation, the findings may have important implications. For example, consistently poor(er) performance observed in hospitalised COVID participants on tasks which emphasise speed could negatively impact daily activities such as driving [42] and may present as a bio-marker for accelerated aging [13]. Given this, frequent follow-ups of COVID-19 survivors, especially those with COVID-19-related hospitalisation and/or long-COVID symptoms, are needed to assess any potential worsening and/or improvement in cognitive function over time. Moreover, remedial interventions, such as mindfulness training, may help reduce cognitive slowing [43] in diverse samples impacted by COVID-19.

## Conclusions

The findings of this follow-up study indicate some cognitive normalisation over a 6-month period in young and middle-aged COVID-19 survivors. However, those participants who had required hospitalisation due to COVID-19, compared to those who did not, continued to display multifaceted cognitive impairment. Continuous follow-up assessments are required to determine whether cognitive improvement continues over time in COVID-19 survivors, particularly in hospitalised/long-COVID participants or whether cognitive function in this sub-group worsens further unless addressed by suitable interventions.

## Supporting information

Vakani et al. supplementary material Vakani et al. supplementary material

## Supplementary material

The supplementary material for this article can be found at https://doi.org/10.1192/j.eurpsy.2024.7.
