# Supplementary material for: Cognitive and mental health trajectories of COVID-19: Role of hospitalisation and long-COVID symptoms
Source: Eur Psychiatry. 2024 Feb 5;67(1):e17. doi: 10.1192/j.eurpsy.2024.7 (PMC10966616; doi:10.1192/j.eurpsy.2024.7)
Supplement: Vakani et al. supplementary material [file S0924933824000075sup001.docx]

Supplementary Table 1. Characteristics of the participants with both T1 and T2 assessments (*n* = 138; current sample) and those with only T1 assessments [*n* = 84 of 222 from Vakani et al. (2023) including 41 COVID and 30 non-COVID participants who did not respond and 13 non-COVID participants who were excluded because of becoming COVID positive between T1 and T2].

|  | | Sample from Vakani et al. (2023)  (N = 222; 129 with and 93 without a COVID history) | | COVID Only Group  (*n* = 129) | |
| --- | --- | --- | --- | --- | --- |
|  |  | T1 & T2  Group  (*n* = 138, current investigation) | T1 Only  Group  (*n* = 84) | T1 & T2  Group  (*n* = 88, current investigation) | T1 Only  Group  (*n* = 41) |
|  |  | Mean (*SD*) | Mean (*SD*) | Mean (*SD*) | Mean (*SD*) |
| Age (Years) | | 39.72±11.81 | 37.83±12.45 | 40.47±10.55 | 41.63±12.56 |
|  |  | *n* (% of Total) | *n* (% of Total) | *n* (% of Total) | *n* (% of Total) |
| Ethnicity | White British | 94 (68.1%) | 54 (64.3%) | 74 (84.1%) | 33 (80.5%) |
|  | South Asian | 29 (21.0%) | 26 (31.0%) | 5 (5.7%) | 8 (19.5%) |
|  | Other Asian | 3 (2.2%) | 2 (2.4%) | 1 (1.1%) | 0 (0%) |
|  | Black British | 1 (0.7%) | 2 (2.4%) | 1 (1.1%) | 0 (0%) |
|  | Mixed Race | 9 (6.5%) | 0 (0%) | 6 (6.8%) | 0 (0%) |
|  | Other | 2 (1.4%) | 0 (0%) | 1 (1.1%) | 0 (0%) |
| Educational Background | High School | 5 (3.6%) | 7 (8.3%) | 3 (3.4%) | 4 (9.8%) |
|  | College/6th Form | 26 (18.8%) | 5 (6.0%) | 19 (21.6%) | 1 (2.4%) |
|  | Vocational Qualification | 12 (8.7%) | 11 (13.1%) | 9 (10.2%) | 7 (17.1%) |
|  | Bachelor's Degree | 45 (32.6%) | 38 (45.2%) | 28 (31.8%) | 22 (53.7%) |
|  | Master's Degree | 38 (27.5%) | 19 (22.6%) | 21 (23.9%) | 5 (12.2%) |
|  | PhD or Higher | 9 (6.5%) | 4 (4.8%) | 5 (5.7%) | 2 (4.9%) |
|  | Prefer not to say | 3 (2.2%) | 0 (0%) | 3 (3.4%) | 0 (0%) |
| Employment Status | Employed Full-time | 69 (50.0%) | 46 (54.8%) | 40 (45.5%) | 21 (51.2%) |
|  | Employed Part-time | 27 (19.6%) | 11 (13.1%) | 19 (21.6%) | 7 (17.1%) |
|  | Student Full-time | 13 (9.4%) | 6 (7.1%) | 7 (8.0%) | 2 (4.9%) |
|  | Student Part-time | 1 (0.7%) | 2 (2.4%) | 0 (0%) | 1 (2.4%) |
|  | Unemployed | 1 (0.7%) | 1 (1.2%) | 1 (1.1%) | 0 (0%) |
|  | Retired | 2 (1.4%) | 2 (2.4%) | 1 (1.1%) | 1 (2.4%) |
|  | Semi-retired | 4 (2.9%) | 2 (2.4%) | 2 (2.3%) | 1 (2.4%) |
|  | Homemaker | 2 (1.4%) | 1 (1.2%) | 2 (2.3%) | 0 (0%) |
|  | Unable to Work | 8 (5.8%) | 6 (7.1%) | 7 (8.0%) | 5 (12.2%) |
|  | Other | 6 (4.3%) | 7 (8.3%) | 5 (5.7%) | 3 (7.3%) |
|  | Prefer not to say | 5 (3.6%) | 0 (0%) | 4 (4.5%) | 0 (0%) |
| Physical Health Conditions | Cancer | 3 (2.2.%) | 2 (2.4%) | 3 (3.4%) | 2 (4.9%) |
|  | Diabetes | 12 (8.7%) | 3 (3.6%) | 7 (8.0%) | 3 (7.3%) |
|  | Heart Condition | 6 (4.3%) | 4 (4.8%) | 4 (4.5%) | 4 (9.8%) |
|  | Immunosuppressed | 7 (5.1%) | 3 (3.6%) | 7 (8.0%) | 1 (2.4%) |
|  | Kidney Disease | 1 (0.7%) | 0 (0%) | 1 (1.1%) | 0 (0%) |
|  | Liver Disease | 0 (0%) | 3 (3.6%) | 0 (0%) | 2 (4.9%) |
|  | Lung Condition | 22 (15.9%) | 13 (15.5%) | 18 (20.5%) | 10 (24.4%) |
|  | Neurological Condition | 5 (3.6%) | 3 (3.6%) | 5 (5.7%) | 2 (4.9%) |
|  | Obesity | 17 (12.3%) | 8 (9.5%) | 12 (13.6%) | 6 (14.6%) |
|  | Organ Transplantation | 1 (0.7%) | 0 (0%) | 1 (1.1%) | 0 (0%) |
| Mental Health Conditions | Anorexia Nervosa | 2 (1.4%) | 2 (2.4%) | 1 (1.1%) | 1 (2.4%) |
|  | Anxiety | 57 (41.3%) | 38 (45.2%) | 38 (43.2%) | 18 (43.9%) |
|  | ADHD | 4 (2.9%) | 2 (2.4%) | 3 (3.4%) | 0 (0%) |
|  | Depression | 47 (34.1%) | 28 (33.3%) | 33 (37.5%) | 15 (36.6%) |
|  | Eating Disorder(s) | 9 (6.5%) | 3 (3.6%) | 7 (8.0%) | 1 (2.4%) |
|  | Insomnia | 26 (18.8%) | 16 (19.0%) | 21 (23.9%) | 8 (19.5%) |
|  | OCD | 6 (4.3%) | 6 (7.1%) | 4 (4.5%) | 3 (7.3%) |
|  | Panic Disorder | 12 (8.7%) | 5 (6.0%) | 7 (8.0%) | 3 (7.3%) |
|  | Personality Disorder | 4 (2.9%) | 1 (1.2%) | 3 (3.4%) | 1 (2.4%) |
|  | Phobias | 12 (8.7%) | 4 (4.8%) | 6 (6.8%) | 3 (7.3%) |
|  | PTSD | 15 (10.9%) | 9 (10.7%) | 12 (13.6%) | 6 (14.6%) |
|  | Psychosis | 2 (1.4%) | 3 (3.6%) | 1 (1.1%) | 1 (2.4%) |
|  | Other | 2 (1.4%) | 1 (1.2%) | 2 (2.3%) | 0 (0%) |

Abbreviations: ADHD, Attention Deficit Hyperactivity Disorder; F, Females; M, Males; OCD, Obsessive Compulsive Disorder; PTSD, Post-Traumatic Stress Disorder.

Supplementary Table 2. Prevalence of COVID-19 symptoms in the COVID participants with both T1 and T2 assessments (*n* = 88 of 129 from Vakani et al.; classified by hospitalisation history) and those with only T1 assessments (*n* = 41 of 129 from Vakani et al who did not respond at T2).

|  | | T1 Only  Group  (*n* = 41) | T1 & T2 Group  (all, *n* = 88) | | T1 & T2 Group, Classified by Hospitalisation History | | | |
| --- | --- | --- | --- | --- | --- | --- | --- | --- |
|  |  |  |  |  | Hospitalised COVID Group  (*n* = 15; 3 M, 12 F) | | Non-hospitalised COVID Group  (*n* = 73; 11 M, 62 F) | |
|  |  |  | T1 | T2 | T1 | T2 | T1 | T2 |
|  |  | *n (*% of Total) | *n (*% of Total) | *n (*% of Total) | *n (*% of Total) | *n (*% of Total) | *n (*% of Total) | *n (*% of Total) |
| Hospitalisation due to COVID | | 5 (12.2%) | 15 (17.0%) | | / | / | / | / |
| Acute COVID-19 Symptoms^a^ | Temperature | 33 (80.5%) | 66 (75.0%) | 66 (79.5%) | 13 (86.7%) | 12 (85.7%) | 53 (72.6%) | 52 (75.4%) |
|  | Dry Cough | 29 (70.7%) | 57 (64.8%) | 57 (68.7%) | 12 (80.0%) | 13 (92.9%) | 45 (61.6%) | 44 (63.8%) |
|  | Loss of Taste and/or Smell | 29 (70.7%) | 54 (61.4%) | 53 (63.9%) | 9 (60.0%) | 10 (71.4%) | 45 (61.6%) | 43 (62.3%) |
|  | Other | 26 (63.4%) | 57 (64.8%) | 38 (45.8%) | 8 (53.3%) | 7 (50.0%) | 49 (67.1%) | 31 (42.5%) |
| Chronic COVID-19 Symptoms  (Long-COVID)^b^ | Abdominal pain | 15 (36.6%) | 34 (38.6%) | 27 (32.9%) | 8 (53.3%) | 4 (28.6%) | 26 (35.6%) | 23 (33.8%) |
|  | Arrhythmia | 21 (51.2%) | 51 (58.0%) | 42 (51.2%) | 11 (73.3%) | 10 (71.4%) | 40 (54.8%) | 32 (47.1%) |
|  | Body chills | 19 (46.3%) | 42 (47.7%) | 28 (34.1%) | 4 (26.7%) | 5 (35.7%) | 38 (52.1%) | 23 (33.8%) |
|  | Breathing problems | 27 (65.9%) | 64 (72.7%) | 52 (63.4%) | 14 (93.3%) | 13 (92.9%) | 50 (68.5%) | 39 (57.4%) |
|  | Chest pain | 19 (46.3%) | 50 (56.8%) | 34 (41.5%) | 12 (80.0%) | 12 (85.7%) | 38 (52.1%) | 22 (32.4%) |
|  | Chilblains | 6 (14.6%) | 13 (14.8%) | 11 (13.4%) | 3 (20.0%) | 3 (21.4%) | 10 (13.7%) | 8 (11.8%) |
|  | Confusion/delirium | 28 (68.3%) | 49 (55.7%) | 51 (62.2%) | 11 (73.3%) | 11 (78.6%) | 38 (52.1%) | 40 (58.8%) |
|  | Diarrhoea | 14 (34.1%) | 30 (34.1%) | 24 (29.3%) | 7 (46.7%) | 6 (42.9%) | 23 (31.5%) | 18 (26.5%) |
|  | Dry cough | 15 (36.6%) | 31 (35.2%) | 31 (37.8%) | 6 (40.0%) | 9 (64.3%) | 25 (34.2%) | 16 (23.5%) |
|  | Exhaustion/fatigue | 34 (82.9%) | 80 (90.9%) | 67 (81.7%) | 15 (100.0%) | 13 (92.9%) | 65 (89.0%) | 54 (79.4%) |
|  | Hallucinations | 9 (22.0%) | 10 (11.4%) | 8 (9.76%) | 2 (13.3%) | 4 (28.6%) | 8 (11.0%) | 4 (5.9%) |
|  | Headaches | 29 (70.7%) | 66 (75.0%) | 56 (68.3%) | 12 (80.0%) | 10 (71.4%) | 54 (74.0%) | 46 (67.6%) |
|  | Insomnia | 31 (75.6%) | 70 (79.5%) | 61 (74.4%) | 14 (93.3%) | 12 (85.7%) | 56 (76.7%) | 49 (72.1%) |
|  | Irritability | 28 (68.3%) | 66 (75.0%) | 57 (69.5%) | 14 (93.3%) | 13 (92.9%) | 52 (71.2%) | 44 (64.7%) |
|  | Lack of appetite | 17 (41.5%) | 45 (51.1%) | 22 (26.8%) | 10 (66.7%) | 5 (35.7%) | 35 (47.9%) | 17 (25.0%) |
|  | Loss of taste and/or smell | 16 (39.0%) | 31 (35.2%) | 22 (26.8%) | 7 (46.7%) | 5 (35.7%) | 24 (32.9%) | 17 (25.0%) |
|  | Mild cognitive problems | 34 (82.9%) | 73 (83.0%) | 68 (82.9%) | 14 (93.3%) | 13 (92.9%) | 59 (80.8%) | 55 (80.9%) |
|  | Muscle/body ache | 29 (70.7%) | 68 (77.3%) | 59 (72.0%) | 12 (80.0%) | 12 (85.7%) | 56 (76.7%) | 47 (69.1%) |
|  | Sore eyes/conjunctivitis | 18 (43.9%) | 41 (46.6%) | 27 (32.9%) | 6 (40.0%) | 7 (50.0%) | 35 (47.9%) | 20 (29.4%) |
|  | Sore throat | 13 (31.7%) | 34 (38.6%) | 35 (42.7%) | 8 (53.3%) | 10 (71.4%) | 26 (35.6%) | 25 (36.8%) |
|  | Temperature | 10 (24.4%) | 26 (29.5%) | 18 (22.0%) | 3 (20.0%) | 4 (28.6%) | 23 (31.5%) | 14 (20.6%) |
|  | Vomiting/nausea | 10 (24.4%) | 24 (27.3%) | 20 (24.4%) | 6 (40.0%) | 5 (35.7%) | 18 (24.7%) | 15 (22.1%) |
|  | Other | 8 (19.5%) | 21 (23.9%) | 18 (22.0%) | 4 (26.7%) | 3 (21.4%) | 17 (23.3%) | 15 (22.1%) |
| Subjective Cognitive Function Impairment^a^ | | 32 (78.0%) | 69 (78.4%) | 66 (79.5%) | 14 (93.3%) | 13 (86.7%) | 55 (75.3%) | 53 (72.6%) |
| Subjective Reduced Psychological Well-being^a^ | | 30 (73.2%) | 70 (79.5%) | 58 (69.9%) | 14 (93.3%) | 12 (80.0%) | 56 (76.7%) | 46 (63.0%) |

Abbreviations: ADHD, Attention Deficit Hyperactivity Disorder; F, Females; M, Males; OCD, Obsessive Compulsive Disorder; PTSD, Post-Traumatic Stress Disorder.

^a^ Data not available for 5 participants at T2 (1 hospitalised, 4 non-hospitalised); ^b^ Data not available for 6 participants at T2 (1 hospitalised, 5 non-hospitalised).

Supplementary Table 3. Cognitive characteristics at T1 of the participants with both T1 and T2 assessments and those with only T1 assessment.

| Measures | | COVID Group | | Non-COVID Group | |
| --- | --- | --- | --- | --- | --- |
|  |  | T1 & T2 Group  (*n* = 88) | T1 Only Group  (*n* = 41) | T1 & T2 Group  (*n* = 50) | T1 Only Group  (*n* = 43) |
|  |  | Mean (*SD*) | Mean (*SD*) | Mean (*SD*) | Mean (*SD*) |
| Cognitive Function | | | | | |
| Processing Speed | Response accuracy (%) | 95.82 (6.27) ^a^ | 95.39 (8.56) ^c^ | 95.78 (7.60) ^b^ | 97.46 (4.09) ^b^ |
|  | RT (correct responses, ms) | 375.89 (80.51) ^a^ | 377.97 (84.60) ^c^ | 354.71 (79.64) ^b^ | 353.29 (66.22) ^b^ |
|  | RT variability (SD of RT) | 88.23 (40.73) ^a^ | 83.54 (44.27) ^c^ | 70.04 (34.67) ^b^ | 74.36 (41.72) ^b^ |
| Attention | Response accuracy (%) | 95.50 (8.65) ^a^ | 94.24 (8.48) ^d^ | 97.71 (4.48) ^c^ | 97.85 (4.91) ^c^ |
|  | RT (correct responses, ms) | 494.45 (94.54) ^a^ | 536.14 (93.67) ^d^ | 463.52 (92.97) ^c^ | 475.73 (103.53) ^c^ |
| Working Memory | Response accuracy (%) | 92.44 (8.48) ^b^ | 91.05 (8.25) ^b^ | 92.96 (7.75) ^c^ | 95.58 (4.64) ^b^ |
| Executive Function | Accuracy (%) | 94.54 (7.46) | 95.27 (6.74) | 95.20 (8.44) | 94.62 (8.81) ^e^ |
|  | Completion time (ms) | 33626.11 (22201.51) | 32239.37 (10144.19) | 29598.04 (9665.89) | 30630.08 (15341.01) ^e^ |
| Memory | Recognition accuracy (%) | 89.95 (9.11) ^b^ | 88.29 (7.20) ^b^ | 92.30 (7.50) | 90.44 (8.97) |
| Mental Health and Well-being | | | | | |
| Mental Health (DASS-21) | Depression | 14.11 (10.50) | 15.17 (10.58) | 9.36 (9.69) | 11.86 (12.15) |
|  | Anxiety | 10.59 (8.75) | 10.20 (8.27) | 7.04 (7.56) | 7.26 (8.53) |
|  | Stress | 14.70 (9.26) | 14.10 (10.01) | 13.28 (10.19) | 12.93 (10.45) |
| Sleep Quality (PSQI) | Global Score | 9.95 (3.70) | 9.80 (3.51) | 6.54 (3.25) | 7.53 (4.33) |

Sample size reduced ^a^ by 8; ^b^ by 1; ^c^ by 2; ^d^ by 5; ^e^ by 3.

Abbreviations: ms, milliseconds; RT, Reaction Time.

Supplementary Table 4. Demographic characteristics of the current sample (N = 138).

|  | | COVID Group  (*n* = 88; 14 M, 74 F) | Non-COVID Group  (*n* = 50; 11 M, 39 F) |
| --- | --- | --- | --- |
|  |  | *n* (% of Total) | *n* (% of Total) |
| Ethnicity | White British | 74 (84.2%) | 20 (40.0%) |
|  | South Asian | 6 (6.8%) | 24 (48.0%) |
|  | Other Asian | 1 (1.1%) | 3 (6.0%) |
|  | Black British | 0 (0%) | 0 (0%) |
|  | Mixed Race | 5 (5.7%) | 3 (6.0%) |
|  | Other | 2 (2.3%) | 0 (0%) |
| Educational Background | High School | 4 (4.5%) | 1 (2.0%) |
|  | College/6th Form | 15 (17.0%) | 8 (16.0%) |
|  | Vocational Qualification | 10 (11.4%) | 1 (2.0%) |
|  | Bachelor's Degree | 29 (33.0%) | 20 (40.0%) |
|  | Master's Degree | 21 (23.9%) | 15 (30.0%) |
|  | PhD or Higher | 5 (5.7%) | 4 (8.0%) |
|  | No Education | 0 (0%) | 1 (2.0%) |
|  | Prefer not to say | 4 (4.5%) | 0 (0%) |
| Employment Status | Employed Full-time | 40 (45.5%) | 30 (60.0%) |
|  | Employed Part-time | 18 (20.5%) | 9 (18.0%) |
|  | Student Full-time | 4 (4.5%) | 5 (10.0%) |
|  | Student Part-time | 1 (1.1%) | 1 (2.0%) |
|  | Unemployed | 1 (1.1%) | 0 (0%) |
|  | Retired | 1 (1.1%) | 2 (4.0%) |
|  | Semi-retired | 1 (1.1%) | 0 (0%) |
|  | Homemaker | 1 (1.1%) | 0 (0%) |
|  | Unable to Work | 12 (13.6%) | 1 (2.0%) |
|  | Other | 7 (8.0%) | 2 (4.0%) |
|  | Prefer not to say | 2 (2.3%) | 0 (0%) |

Abbreviations: F, Females; M, Males.

Supplementary Table 5. T1 characteristics of COVID group participants, classified by hospitalisation history, separately for participants with both T1 and T2 assessments (current sample) or only T1 assessment.

|  | | T1 and T2 COVID group  (*n* = 88) | | T1 only COVID Group  (*n* = 41) | |
| --- | --- | --- | --- | --- | --- |
|  |  | Hospitalised  (*n* = 15; 3 M, 12 F) | Non-hospitalised  (*n* = 73; 11 M, 62 F) | Hospitalised  (*n* = 5; 1 M, 4 F) | Non-hospitalised  (*n* = 36; 8 M, 28 F) |
|  |  | Mean (*SD*) | Mean (*SD*) | Mean (*SD*) | Mean (*SD*) |
| Age (Years) |  | 45.20±10.53 | 40.10±10.26 | 42.60±15.13 | 41.50±12.41 |
|  |  | *n* (% of Total) | *n* (% of Total) | *n* (% of Total) | *n* (% of Total) |
| Ethnicity | White British | 14 (93.3%) | 60 (82.2%) | 5 (100.0%) | 28 (77.8%) |
|  | South Asian | 0 (0%) | 5 (6.8%) | 0 (0%) | 8 (22.2%) |
|  | Other Asian | 0 (0%) | 1 (1.4%) | 0 (0%) | 0 (0%) |
|  | Black British | 0 (0%) | 1 (1.4%) | 0 (0%) | 0 (0%) |
|  | Mixed Race | 1 (6.7%) | 5 (6.8%) | 0 (0%) | 0 (0%) |
|  | Other | 0 (0%) | 1 (1.4%) | 0 (0%) | 0 (0%) |
| Educational Background | High School | 1 (6.7%) | 2 (2.7%) | 2 (40.0%) | 2 (5.6%) |
|  | College/6th Form | 2 (13.3%) | 17 (23.3%) | 0 (0%) | 1 (2.8%) |
|  | Vocational Qualification | 4 (26.7%) | 5 (6.8%) | 1 (20.0%) | 6 (16.7%) |
|  | Bachelor's Degree | 5 (33.3%) | 23 (31.5%) | 2 (40.0%) | 20 (55.6%) |
|  | Master's Degree | 3 (20.0%) | 18 (24.7%) | 0 (0%) | 5 (13.9%) |
|  | PhD or Higher | 0 (0%) | 5 (6.8%) | 0 (0%) | 2 (5.6%) |
|  | No Education | 0 (0%) | 0 (0%) | 0 (0%) | 0 (0%) |
|  | Prefer not to say | 0 (0%) | 3 (4.1%) | 0 (0%) | 0 (0%) |
| Employment Status | Employed Full-time | 6 (40.0%) | 34 (46.6%) | 3 (60.0%) | 18 (50.0%) |
|  | Employed Part-time | 6 (40.0%) | 13 (17.8%) | 1 (20.0%) | 6 (16.7%) |
|  | Student Full-time | 1 (6.7%) | 6 (8.2%) | 0 (0%) | 2 (5.6%) |
|  | Student Part-time | 0 (0%) | 0 (0%) | 0 (0%) | 1 (2.8%) |
|  | Unemployed | 0 (0%) | 1 (1.4%) | 0 (0%) | 0 (0%) |
|  | Retired | 0 (0%) | 1 (1.4%) | 0 (0%) | 1 (2.8%) |
|  | Semi-retired | 0 (0%) | 2 (2.7%) | 0 (0%) | 1 (2.8%) |
|  | Homemaker | 1 (6.7%) | 1 (1.4%) | 0 (0%) | 0 (0%) |
|  | Unable to Work | 1 (6.7%) | 6 (8.2%) | 1 (20.0%) | 4 (11.1%) |
|  | Other | 0 (0%) | 5 (6.8%) | 0 (0%) | 3 (8.3%) |
|  | Prefer not to say | 0 (0%) | 4 (5.5%) | 0 (0%) | 0 (0%) |
| Physical Health Conditions | Cancer | 0 (0%) | 3 (4.1%) | 0 (0%) | 2 (5.6%) |
|  | Diabetes | 1 (6.7%) | 6 (8.2%) | 1 (20.0%) | 2 (5.6%) |
|  | Heart Condition | 1 (6.7%) | 3 (4.1%) | 1 (20.0%) | 3 (8.3%) |
|  | Immunosuppressed | 2 (13.3%) | 5 (6.8%) | 0 (0%) | 1 (2.8%) |
|  | Kidney Disease | 0 (0%) | 1 (1.4%) | 0 (0%) | 0 (0%) |
|  | Liver Disease | 0 (0%) | 0 (0%) | 1 (20.0%) | 1 (2.8%) |
|  | Lung Condition | 6 (40.0%) | 12 (16.4%) | 2 (40.0%) | 8 (22.2%) |
|  | Neurological Condition | 2 (13.3%) | 3 (4.1%) | 0 (0%) | 2 (5.6%) |
|  | Obesity | 1 (6.7%) | 11 (15.1%) | 2 (40.0%) | 4 (11.1%) |
|  | Organ Transplantation | 0 (0%) | 1 (1.4%) | 0 (0%) | 0 (0%) |
| Mental Health Conditions | Anorexia Nervosa | 1 (6.7%) | 0 (0%) | 0 (0%) | 1 (2.8%) |
|  | Anxiety | 5 (33.3%) | 33 (45.2%) | 1 (20.0%) | 17 (47.2%) |
|  | ADHD | 0 (0%) | 3 (4.1%) | 0 (0%) | 0 (0%) |
|  | Depression | 5 (33.3%) | 28 (38.4%) | 1 (20.0%) | 14 (38.9%) |
|  | Eating Disorder(s) | 1 (6.7%) | 6 (8.2%) | 0 (0%) | 1 (2.8%) |
|  | Insomnia | 3 (20.0%) | 18 (24.7%) | 0 (0%) | 8 (22.2%) |
|  | OCD | 1 (6.7%) | 3 (4.1%) | 0 (0%) | 3 (8.3%) |
|  | Panic Disorder | 0 (0%) | 7 (9.6%) | 0 (0%) | 3 (8.3%) |
|  | Personality Disorder | 1 (6.7%) | 2 (2.7%) | 0 (0%) | 1 (2.8%) |
|  | Phobias | 1 (6.7%) | 5 (6.8%) | 0 (0%) | 3 (8.3%) |
|  | PTSD | 2 (13.3%) | 10 (13.7%) | 1 (20.0%) | 5 (13.9%) |
|  | Psychosis | 0 (0%) | 1 (1.4%) | 0 (0%) | 1 (2.8%) |
|  | Other | 0 (0%) | 2 (2.7%) | 0 (0%) | 0 (0%) |

Abbreviations: F, Females; M, Males.

Supplementary Table 6. Associations (Pearson’s *r*) between the changes in cognitive function and mental health measures.

|  | Processing Speed  (*n* = 128) | | | Attention  (*n* = 125) | | Working Memory  (*n* = 134) | Executive Function  (*n* = 136) | | Memory  (*n* = 137) |
| --- | --- | --- | --- | --- | --- | --- | --- | --- | --- |
|  | Response accuracy (%) | RT correct responses (ms) | RT variability  (SD of RT) | Response accuracy (%) | RT correct responses (ms) | Accuracy (%) | Response accuracy (%) | Completion time (ms) | Recognition accuracy (%) |
|  | *r (p)* | *r (p)* | *r (p)* | *r (p)* | *r (p)* | *r (p)* | *r (p)* | *r (p)* | *r (p)* |
| Mental Health (DASS-21) | | | | | | | | | |
| Depression | 0.11 (0.20) | -0.02 (0.87) | 0.06 (0.50) | 0.004 (0.97) | -0.17 (0.07) | 0.07 (0.45) | 0.11 (0.22) | 0.04 (0.66) | 0.03 (0.72) |
| Anxiety | 0.08 (0.40) | -0.06 (0.51) | -0.01 (0.94) | 0.14 (0.12) | -0.03 (0.77) | 0.06 (0.53) | 0.12 (0.18) | -0.11 (0.21) | 0.12 (0.18) |
| Stress | -0.01 (0.94) | -0.04 (0.70) | -0.04 (0.69) | 0.11 (0.21) | -0.04 (0.63) | 0.05 (0.56) | 0.10 (0.23) | -0.02 (0.79) | -0.02 (0.81) |
| Sleep Quality (PSQI) | | | | | | | | | |
| Global Score | 0.03 (0.70) | -0.02 (0.87) | -0.02 (0.83) | 0.01 (0.90) | -0.03 (0.78) | -0.08 (0.39) | -0.01 (0.93) | -0.02 (0.81) | 0.19 **(0.03)** |
